# To Get Leeches to Fasten

**Published:** 1879-08

**Authors:** 


### TO GET LEECHES TO FASTEN.

Almost every physician has at times experienced the difficulty of getting these animals to bite. The following plan is commended, and will be found effectual in all cases when the leeches are healthy. Put the animals in a small glass vessel half filled with cold water. The part of the body which is to receive them is carefully washed with warm water, and the glass is quickly inverted upon the skin. The leeches attach themselves with surprising rapidity. When all the animals have bitten the glass is carefully removed, the water escaping being absorbed by a sponge. If a single leech is to be applied, the same plan is adopted, using a test tube in place of a glass; by this means the animal may be compelled to bite at just the point desired.

